# A review on impact of adipocytokines in post postmenopausal women

**DOI:** 10.6026/973206300211906

**Published:** 2025-07-31

**Authors:** Eslavath Sukanya, Ponnazhagan, Veluri Ganesh

**Affiliations:** 1 Department of Biochemistry, Sri Chamundeshwari Medical College, Hospital & Research Institute, Ramanagara, Channapatna, Karnataka, India; 2 Department of Biochemistry, Meenakshi Medical College Hospital and Research Institute, Enathur, Kanchipuram, Tamil Nadu, India; 3 Department of Biochemistry, PES University Institute of Medical Sciences and Research, Electronic City, Bengaluru, Karnataka, India

**Keywords:** Adiponectin, AMPK-activated protein kinase, ghrelin, insulin resistance, leptin, Vitamin D

## Abstract

Obesity in postmenopausal women is a major global health concern due to hormonal and metabolic changes. This review explores the roles of ghrelin, leptin, adiponectin, insulin, AMPK, and vitamin D in energy regulation and weight fluctuation. Estrogen decline post-menopause alters metabolic balance, contributing to fat accumulation and insulin resistance. Reduced adiponectin and disrupted leptin-ghrelin signalling are linked with increased body mass index. Vitamin D sufficiency, alongside lifestyle changes, may support weight management in this population.

## Background:

The postmenopausal phase is marked by significant physiological and hormonal changes, which influence weight fluctuations and increase the risk of obesity. Menopause is a transitional period that typically begins 1-2 years before the cessation of menstruation, known as the peri-menopausal phase. It generally occurs between the ages of 45 and 55, though some individuals may experience it earlier. During this time, many women experience an average weight gain of 2 to 2.5 kg over three years, particularly in the abdominal region, leading to an increased risk of metabolic disorders such as insulin resistance, type 2 diabetes, and cardiovascular diseases [1-2]. Notably, this weight gain is comparable to that observed in age-matched premenopausal women [3]. While estrogen decline is a key contributor, the molecular mechanisms underlying menopausal weight fluctuations involve complex interactions among multiple hormones and metabolic regulators. Among the key players in this molecular network are ghrelin, leptin, adiponectin, insulin, AMPK-activated protein kinase (AMPK), and vitamin D. Ghrelin, commonly known as the "hunger hormone," has a crucial function in promoting appetite and promoting fat storage [4]. However, leptin, acts as a satiety signal, helping to regulate energy balance by inhibiting hunger. The hormone adiponectin, which is released by adipose tissue, improves insulin sensitivity, and has anti-inflammatory properties, making it crucial for maintaining metabolic health [5]. Insulin, a hormone central to glucose metabolism, often becomes dysregulated during menopause, leading to insulin resistance and associated weight gain [6]. The AMPK, an energy-sensing enzyme, is essential for the maintenance of energy homeostasis by facilitating the intake of glucose and fatty acid oxidation [7]. Vitamin D, traditionally recognized for its role in bone health, has also been implicated in metabolic processes, including insulin sensitivity and inflammation. Understanding the intricate relationships among these molecules is essential for developing effective strategies to manage weight fluctuations in postmenopausal women [8]. This review aims to explore the molecular connections between ghrelin, leptin, adiponectin, insulin, AMPK, and vitamin D, and their collective impact on weight regulation in overweight postmenopausal women. By analyzing current research, this review will provide insights into potential therapeutic targets for managing weight and metabolic health during this critical stage of life. Therefore, it is of interest to describe the molecular interplay among ghrelin, leptin, adiponectin, insulin, AMPK, and vitamin D in relation to weight regulation in overweight postmenopausal women.

## Key factors for weight fluctuations among Postmenopausal women:

After menopause, significant hormonal changes occur, particularly a decline in estrogen, which is crucial for regulating body weight, fat distribution, and metabolism. Decreased estrogen levels are linked to higher abdominal adiposity, decreased energy expenditure, and insulin resistance [9]. The progesterone levels also drop, leading to weight gain, memory loss, and mood disturbances [3]. The testosterone, though primarily a male hormone, also declines in women, contributing to reduced libido and fatigue [10]. In addition to hormonal changes, postmenopausal women often experience chronic low-grade inflammation, with elevated cytokines like IL-6 and TNF-α, which promote resistance to insulin and weight gain [11]. A decrease in basal metabolic rate (BMR) and mitochondrial efficiency, combined with genetic factors such as differential gene expression and epigenetic modifications, further contribute to metabolic dysregulation and weight gain. Gut microbiome alterations and stress-induced cortisol elevation are also linked to increased abdominal fat. Additionally, bone degeneration, or osteopenic sarco obesity, is common, though a healthy lifestyle may mitigate its severity. These interconnected factors underscore the complexity of weight management in postmenopausal women and the importance of a holistic approach that addresses hormonal, metabolic, and lifestyle factors. Below is the schematic representation of key factors involved in weight fluctuations in postmenopausal women (Figure 1 - see PDF).

## Role of ghrelin in postmenopausal weight gain:

Ghrelin, often termed the "hunger hormone," plays a crucial role in regulating appetite and energy balance, making it a key factor in postmenopausal obesity [12]. Primarily secreted by the stomach, ghrelin stimulates hunger and promotes fat storage. In postmenopausal women, declining estrogen levels can disrupt ghrelin regulation, leading to increased appetite and abdominal weight gain [13-14]. An association was seen between changes in ghrelin levels during perimenopause and changes in other adipokines, including adiponectin, in comparison to the pre-and postmenopause periods [15]. Ghrelin not only affects energy intake but also interacts with other hormones like leptin and insulin, which are crucial for maintaining energy balance. The reduced sensitivity to these hormones after menopause exacerbates metabolic disturbances, increasing fat deposition and insulin resistance. The Figure 2 (see PDF) illustrates how declining estrogen levels influence ghrelin and leptin regulation, contributing to postmenopausal weight gain. The Karim *et al.* stated that postmenopausal women greater than 10 years into menopause had significantly lower concentrations of ghrelin compared with women within 6 years of menopause, which can partially explain the increased risk of stroke and cardiovascular diseases in this population among older postmenopausal women [16].

## Leptin and adiponectin in postmenopausal obesity:

Leptin (167-amino acid polypeptide) hormone released by the fat tissue (adipocytes) and crucial for the regulation of body weight [17]. The ob gene makes this hormone, which has a 21-amino acid N-terminal secretory-signal sequence. Due to the fact that ob/ob mice, which don't have leptin, are overweight, it was first thought that it might help maintain a healthy weight. Several studies show a connection between the amount of leptin in the body, the body mass index (BMI), and fat storage [18-19]. However, many women in this demographic may experiences leptin resistance, where the body does not respond effectively to leptin's appetite-suppressing signals. This resistance can lead to challenges in weight management, as leptin's satiating effects are diminished. The synthesis of leptin is correlated with the concentration of insulin and rises following the delivery of insulin [20].

Adiponectin is a 30-kDa protein that is mostly made by adipocytes and cardiomyocytes. Its main job is to stimulate insulin sensitivity by increasing fatty acid oxidation in fat tissue and lowering fatty acid levels in the bloodstream and triglyceride (TG) levels inside cells in the liver and muscle [21-22]. Adiponectin constitutes around 0.01% of the total plasma proteins, ranging from 5 to 10 µg/mL. Comparative data indicates that the plasma concentration of adiponectin is higher in women than in men [23]. Impaired levels of circulating adiponectin in obese persons are suggested to have a significant role in the development of cardiovascular and atherosclerosis disorders linked to weight gain and metabolic syndrome [24]. Suboptimal levels of adiponectin in postmenopausal women are frequently associated with insulin resistance and obesity. Elevated levels of adiponectin are linked to enhanced metabolic parameters and may have a protective effect against obesity-related illnesses like type 2 diabetes and cardiovascular disorders [25]. Both leptin and adiponectin have been found to exhibit opposing effects on metabolic processes (Figure 3 - see PDF). While leptin acts to promote energy expenditure, adiponectin is involved in increasing insulin sensitivity and exerting anti-inflammatory effects. The imbalance between these two hormones can exacerbate metabolic derangements in postmenopausal women, leading to increased obesity risk and related complications. For instance, an unfavourable adiponectin to leptin ratio has emerged as a potential marker for increased risk of obesity-related health issues, including breast cancer [26]. The current section primarily presents leptin resistance as a well-established phenomenon in postmenopausal women without acknowledging conflicting research. Some studies indicate that while leptin levels are indeed elevated in obese postmenopausal women, they do not always correspond to resistance. Instead, certain findings suggest that high leptin levels might still exert regulatory effects on appetite and metabolism, albeit in a modified or context-dependent manner. By not addressing these alternative perspectives, the section presents leptin resistance as a uniform outcome, which may not reflect the full scientific discourse.

## Role of insulin in postmenopausal obesity:

Insulin is critical for glucose metabolism, and its dysregulation can significantly impact weight [27]. In postmenopausal women, declining estrogen levels are associated with increased insulin resistance, which can exacerbate weight gain. Hyperinsulinemia, or elevated insulin levels, promotes fat storage, and inhibits lipolysis (the breakdown of fat), contributing to difficulty in weight management [28]. In addition to the absence of progesterone, the hypoestrogenic condition also contributes to the deterioration of metabolic functioning. As women proceed from the "perimenopause" to the "postmenopausal" phase, the presence of hypoestrogenism worsens insulin resistance. This resistance is further exacerbated by the steady and continual increase in cortisol levels that is typical of the ageing process [29]. Generally, it is recognised that cortisol exerts the production of glucose and, as a result, further increases resistance to insulin regulation. Concurrently, hypoestrogenism also partially triggers a notable decrease in growth hormone (GH), which increases the tendency to accumulate abdominal fat and reduces lipid metabolism (Figure 4 - see PDF) [30]. Studies suggest that transdermal estrogen therapy improves glucose homeostasis and reduces the risk of type 2 diabetes in postmenopausal women. The Women's Health Initiative (WHI) trial found that estrogen-alone therapy was associated with a reduced incidence of diabetes in women with prior hysterectomy. Research indicates that HRT may counteract cortisol-induced insulin resistance, which the manuscript acknowledges as a significant contributor to metabolic dysfunction [31].

## AMPK in postmenopausal obesity:

Adenosine monophosphate activated protein kinase (AMPK) is a key energy monitor that regulates metabolic pathways, including those associated with fat oxidation and energy homeostasis. In postmenopausal women, AMPK activity may be reduced during exercise, limiting its effectiveness in promoting fat breakdown and energy expenditure. The regulation of AMPK is heavily influenced by hormonal changes during menopause, particularly the decline in estradiol levels, which is crucial for maintaining energy balance [13]. Regulation of AMPK occurs by the phosphorylation of Thr^172^ by kinases located upstream [32]. As illustrated in Figure 5 (see PDF), AMPK plays a critical role in regulating fat metabolism by enhancing fatty acid oxidation and inhibiting lipogenesis. The decline in estrogen levels during menopause reduces AMPK activation, leading to increased fat storage and insulin resistance [33-34]. In addition, AMPK not only regulates metabolic pathways but also modulates inflammatory cascades to elevate chronic impaired inflammatory diseases such as atherosclerosis [35]. Hence, pharmaceutical therapies aimed at stimulating the AMPK pathway show significant promise in the management of weight gain and metabolic diseases. Several pharmacological compounds have been investigated for their role in stimulating AMPK activity, which could be beneficial in managing postmenopausal obesity and insulin resistance. Metformin has been widely studied in postmenopausal women, showing beneficial effects on weight regulation and metabolic parameters. Resveratrol has been investigated for its potential anti-obesity and anti-diabetic effects in postmenopausal women. Studies show that resveratrol supplementation enhances AMPK activation in skeletal muscle, improving metabolic function.

## Vitamin D in weight gain in postmenopausal women:

The potential role of vitamin D in weight management has garnered significant attention, particularly in the context of postmenopausal obesity. Studies indicate that vitamin D supplementation may facilitate weight loss among overweight postmenopausal women, especially when serum 25-hydroxyvitamin D (25(OH)D) levels reach the adequate threshold. The metabolic benefits of vitamin D are likely mediated through its regulatory effects on adiponectin and inflammatory pathways. Although often classified as a vitamin, vitamin D functions as an active circulating pre-hormone with widespread endocrine effects [36]. The global prevalence of both obesity and vitamin D deficiency has reached epidemic proportions, leading to a surge in research exploring their interrelationship. One pivotal study by Wortsman *et al.* provided compelling evidence that vitamin D, a fat-soluble molecule, can be sequestered in adipose tissue, potentially reducing its bioavailability in individuals with obesity [37]. Additionally, findings from the Women's Health Initiative suggest that combined supplementation of calcium and vitamin D^3^ may help mitigate postmenopausal weight gain [38-39]. Moreover, 25(OH) D, the primary biomarker of vitamin D status, has been inversely associated with weight gain in multiple studies [40-41]. Beyond its role in calcium homeostasis, vitamin D functions as an acute-phase reactant, modulating inflammatory responses in adipose tissue. Increasing 25(OH)D levels may contribute to weight loss by reducing chronic low-grade inflammation-a key factor in metabolic dysfunction. Some evidence suggests that higher doses of vitamin D supplementation (*e.g.*, ≥4000 IU/day) might confer greater metabolic benefits in postmenopausal women, potentially enhancing insulin sensitivity and fat metabolism [42- 43].

Furthermore, 1, 25-dihydroxyvitamin D [1, 25(OH)_2_D], the biologically active form of vitamin D, has been shown to upregulate the expression of key metabolic and inflammatory markers, including leptin, adiponectin, tumor necrosis factor-alpha (TNF-α), transforming growth factor (TGF)-β1, plasminogen activator inhibitor-1, and resisting in visceral adipose tissue. Additionally, 1,25(OH)_2_D inhibits proinflammatory cytokines such as interleukin (IL)-1β, IL-6, IL-8, IL-12, as well as vascular endothelial growth factor (VEGF), C-reactive protein (CRP), nuclear factor kappa B (NF-κB), and mitogen-activated protein kinase (MAPK) signaling pathways. It also reduces toll-like receptor expression, which plays a critical role in immune-mediated inflammation (Figure 6 - see PDF). Conversely, vitamin D promotes the production of anti-inflammatory cytokines, including IL-4, IL-10, and IL-13, while facilitating macrophage transformation towards an anti-inflammatory phenotype [44, 45-46]. Through these mechanisms, vitamin D exerts potent anti-inflammatory effects, which may aid in weight regulation and metabolic health in postmenopausal women. The Table 1 (see PDF) provides a summary of these key hormones, outlining their primary functions, interactions with other metabolic regulators, and clinical implications in the context of postmenopausal obesity.

## Conclusion:

The complex interplay between ghrelin, leptin, adiponectin, insulin, AMPK, and vitamin D in regulating weight during post menopause is shown. Hormonal imbalance due to menopause significantly affects appetite, metabolism, and fat distribution. Thus, targeting these pathways may help improve weight management and reduce metabolic risk in postmenopausal women.
